# Ethnic density and area-deprivation effects on central obesity among ethnic minority people in England—A cross-sectional, multi-level analysis

**DOI:** 10.3389/fepid.2022.1000155

**Published:** 2022-10-05

**Authors:** Vanessa Higgins

**Affiliations:** Cathie Marsh Institute, University of Manchester, Manchester, United Kingdom

**Keywords:** obesity, ethnicity, waist circumference, health inequalities, area deprivation

## Abstract

**Background:**

Central obesity is a risk factor for many health conditions, and it disproportionately affects some ethnic minority groups. Research has shown that there is an association between area environments and obesity, but no studies have explored the association between co-ethnic density and central obesity in the UK (United Kingdom). This paper addresses the following research question: Does the relationship between co-ethnic density/area deprivation and waist circumference differ by ethnic group in England?

**Methods:**

Data come from 4 years of the cross-sectional Health Survey for England (1998, 1999, 2003, 2004) and linked area-level data from the 2001 Census. More recent data on objectively measured central obesity for a nationally representative sample of ethnic minorities does not exist. Multi-level modeling methods account for individual-level and area-level factors. Interaction models test the effect of area deprivation and co-ethnic density for each ethnic group compared with the White reference group.

**Results:**

For women, the relationship between area deprivation and waist circumference does not vary by ethnic group. For Indian and Bangladeshi men there is a decrease in waist circumference as area deprivation increases. There is an increase in waist circumference as co-ethnic density increases for Black Caribbean women. For Indian men there is a decrease in waist circumference as co-ethnic density increases.

**Conclusions:**

Further research is needed to understand the mechanisms through which (1) increasing area-deprivation is protective for Indian and Bangladeshi men and (2) increasing co-ethnic density is associated with an increase in waist circumference for Black Caribbean women but a decrease in waist circumference among Indian men. Each of these results are important because (1) Indian and Bangladeshi men have an increased risk of the metabolic syndrome, which is linked to central obesity, and (2) Black Caribbean women have a higher risk of central obesity than the general population in England.

## Introduction

### Central obesity and ethnicity

Central obesity is a risk factor for Type II diabetes, coronary heart disease and insulin resistance (1). Measures of central obesity are especially important for South Asian ethnic groups who have an increased risk of developing the metabolic syndrome (a group of risk factors for heart disease and other health problems such as diabetes) (2, 3).

Bangladeshi women, Pakistani men and women, Black African women and Black Caribbean women in England have a higher risk of central obesity than the general population in England (4). Recent research found that, after adjusting for a wide range of potential explanatory variables, the pathways to ethnic differences in waist circumference are multi-dimensional with migration, socio-economic inequality and cultural factors each associated with ethnic differences in waist circumference (3).

### Area environments, obesity and ethnicity

The area environment is an important factor that shapes the lives and opportunities of individuals (5, 6). Research has shown that there is an association between area environments and obesity within the UK (7–10). Access to physical activity facilities and fast-food outlets influence individual exposure to an obesogenic environment, while area-level residential deprivation is associated with increased levels of obesity in the UK (8–12). Higher perceptions of social support and social capital in an area are associated with lower levels of obesity (13) while fear of crime and neighborhood disorder are associated with higher levels of obesity (8, 14, 15). Bangladeshi, Pakistani, Black Caribbean and Black African groups are overrepresented in the most deprived neighborhoods in England and Wales (16). Deprived areas have high crime rates and ethnic minority groups worry more about crime than the White majority group in the UK (17–19). Fast-food outlets are also more prevalent in deprived areas (20). However, there is also evidence to suggest that greater proximity to fast food outlets is associated with decreased obesity among South Asian women, suggesting that the obesogenic aspects of deprived neighborhoods may have different effects upon different ethnic groups (21).

A large body of work in the UK has focused on the protective effect of area-level co-ethnic density on ethnic inequalities in health, i.e., that people from ethnic minority groups who live in areas with higher concentrations of people from the same ethnic group have more positive health outcomes than those living in areas with lower levels of co-ethnics, once the effects of associated area deprivation are taken into account (22–24). Studies in the US have explored the relationship between co-ethnic density and weight status, but they have not explored the relationship between co-ethnic density and *central* obesity (25–28). Within the UK, there is only one study (29) that has explored the relationship between co-ethnic density and BMI obesity; the study population was five ethnic groups in East London. The study reported that increased co-ethnic density was associated with a decrease in BMI for Indian women but an increase in BMI for Black African men and women.

This paper addresses the following research question: Does the relationship between co-ethnic density/area deprivation and waist circumference differ by ethnic group in England? This research is novel because there have been no studies in England that have explored the relationship between *central obesity*, and co-ethnic density/area-level deprivation for different ethnic groups. This research, therefore, builds on the previous body of knowledge on ethnic differences in obesity and, particularly, the BMI results from an East London cohort of five ethnic groups (29).

## Methods

### Data

Data from the Health Survey for England (1998, 1999, 2003 and 2004) and the 2001 Census were combined (30–34). These are the most up to date nationally representative data available; there have been no UK studies since that study *central obesity* (rather than BMI) and have a nationally representative sample of ethnic minorities.

The HSE is an annual, cross-sectional survey which provides a nationally representative sample of the population living in private households in England *via* a multi-stage, stratified, probability sample. It is designed to provide information on a wide variety of aspects of the nation's health. Data were obtained *via* a face-to-face interview with a trained interviewer, followed by a nurse visit to the household to take measurements and blood samples. The 1999 and 2004 surveys focus on the health of ethnic minority groups and over-sample Black Caribbean, Indian, Pakistani, Bangladeshi, Irish, Chinese and (in 2004 only) Black African participants (4). The White sample was drawn from the 1998 and 2003 HSE. More detailed information on the methodology of the HSE is available elsewhere (35). The data owners approved a data request to link area-level data on ethnic density from the 2001 Census to the individual-level HSE data, using deterministic linkage based on the individual's residential postcode. In order to minimize disclosure risk, the lowest geographical level of Census data that the data owner would release was at the Middle Super Output Areas (MSOA) level. MSOAs are geographical units, with an average population size of 7,500 individuals, that were created by the ONS so that local area statistics could be produced and nationwide comparisons could be made. The dataset provided by the data owner contained the individual serial numbers of the respondents in the HSE and the MSOA data. This enabled safe deterministic linkage of the MSOA level ethnic density data without revealing respondents' postcodes to the researcher.

### Ethical approval and informed consent

This is secondary data analysis based on the Health Survey for England (HSE) and Census data. These data are reviewed by an independent Research Ethics Committee. Ethical approval was obtained as follows:

- 1998 HSE survey: North Thames Multi-Centre Research Ethics Committee (MREC) and from all Local Research Ethics Committees (LRECs) in England. Approval for the revised blood protocol for minors was obtained from LRECs covering the Primary Sampling Units in which the protocol was adopted.
- 1999 HSE survey: North Thames Multi-Centre Research Ethics Committee and from all Local Research Ethics Committees (LRECs) in England.
- 2003 and 2004 HSE surveys: London Multi-centre Research Ethics Committee (MREC). All relevant Local Research Ethics Committees were informed about the survey.

Informed verbal consent was obtained from participants and documented by the survey field interviewers. The data do not include minors. All data were fully anonymized before they were accessed from the UK Data Service.

### Outcome variable

Waist circumference is a widely used measure of central obesity. WHO recommended thresholds for “increased” waist circumference are intended for diagnostic purposes, including the monitoring of population health. For investigations of etiology, the use of continuous waist circumference is preferable to a categorical outcome (2, 36, 37). This research uses continuous waist circumference as the outcome variable, with continuous hip circumference measurement used as a control variable to account for body size. However, it should be noted that the use of a continuous outcome for obesity does not measure obesity *per se*. The measurement of waist and hip circumference took place during the nurse visit to the household using a tape measure. Waist circumference is measured at the midpoint between the top of the hip bone and the lower rib. Hip circumference is measured below the top of the hip bone at the widest circumference around the buttocks. Waist and hip are each measured twice (and a third time if there are inaccuracies of more than 3 cm between the first and second measurement). The valid mean of each measurement is used in the analyses. More detailed information on these measurements is available elsewhere (35).

### Inclusion criteria

The inclusion criteria for this study were respondents age 16–74 with valid measurements for waist circumference and hip circumference.

### Ethnicity

Ethnicity is a multi-dimensional concept that reflects an expression of belonging based on one of more shared characteristics such as common ancestry, country-of birth, nationality, religion, culture, color or language (38–40). The ethnicity variable within the Health Survey for England data was based on the respondent's perceived ethnic identity and perceived family origins, with slight variation in the questions asked across the four waves of data collection. The ethnic group categories are: Black Caribbean, Black African, Indian, Pakistani, Bangladeshi, Chinese, Irish and White. All ethnic minority groups, apart from the Irish, were as defined in the 2001 Census. The Irish group often identify as White British, so respondents were asked for their mother and father's origins; people were included as being of Irish origin if they were born in Ireland, or their father or mother were born there. The White category includes White British and White Other.

### Area level variables

Co-ethnic density is measured using a continuous variable of the percentage of co-ethnics in an area (Middle Super Output Area). This variable, available from the Census data, was matched onto the HSE dataset. Co-ethnic density was calculated by dividing the number of residents within the area from an individual's own ethnic group by the total number of residents in that area. Area deprivation is measured using the Index of Multiple Deprivation (IMD) 2004 variable. The IMD2004 is a ranking of area according to levels of deprivation based on 2001 Census Lower Super Output Areas (LSOAs). LSOAs are geographical units created by the Office for National Statistics, with an average population size of 1,500 individuals. The IMD 2004 measures deprivation across seven items including income, employment, health and disability, education, skills and training, barriers to housing and services, crime and disorder, and living environment. IMD2004 scores of all LSOAs in England were grouped into quintiles–the first quintile contains the least deprived areas and the fifth quintile contains the most deprived areas. Households within the HSE datasets were then allocated to an IMD2004 quintile according to their postcode. Each individual in a household is assigned to the deprivation quintile of their household. A continuous IMD variable was also created–this was imputed from the categorical IMD variable by assigning the mean value of each IMD category (IMD1-5) to each case within that IMD category.

### Individual level control variables

The following individual-level variables are included as controls in the model as there is evidence that each of these characteristics has an association with obesity (3, 4, 41–49).

- Gender: men; women. All analyses are stratified by gender as previous research highlights gender differences in obesity within ethnic groups.
- Hip: continuous hip circumference measurement (cm).
- Age: included as a continuous variable in the models (defined as age at last birthday). This ensures that the models are corrected for differences in age distributions between ethnic groups.
- Registrar General Social Class based on self-reported occupation: professional; managerial/technical or skilled non-manual; manual (includes skilled, semi-skilled and unskilled manual); never worked; other (includes full-time students if never worked; armed forces; insufficient information).
- Highest educational qualification: degree level or equivalent; higher education below degree; A level or equivalent; GCSE or equivalent and foreign/other; no qualifications; full-time student.
- Equivalised household income quintiles: a measure of income that takes account for the number of people living in the household. A separate category has been derived for ‘missing income data' because 14% of respondents have missing income data. This is a household-level variable that is applied to each member of the household.
- Migration status: derived from two variables to determine (1) whether an individual is UK born or born outside the UK and (2) for those born outside the UK, the length of time that they have lived in the UK. Those aged 16 or over when they migrated to the UK are defined as adult migrants and those aged under 16 when they migrated to the UK are defined as child migrants. Those born in England, Scotland, Wales or Northern Ireland are defined as UK born. Those who were born outside the UK are asked for the year of their migration. This data was combined with year of birth to establish the length of time since immigration for each individual in the dataset. Those who identified as White were not asked which country there were born in, so all White cases were imputed as ‘UK born'.
- English language proficiency: does not read or speak English; reads or speaks English; reads and speaks English. Those who identified as White were not asked about their English Language proficiency so all White cases were imputed as ‘read and speaks English'.
- Dietary fat intake: low (less than 83g/day); medium (83g-122g/day); high (more than 122g/day).
- Physical activity level (moderate or vigorous activity in the last 4 weeks): low (3 or fewer occasions); medium (4-19 occasions); high (20 or more occasions). These levels are based on the UK Government's definition of moderate or vigorous activity and weekly recommendations for adults at the time the surveys were conducted, i.e. at least 30 minutes of moderate activity on a regular basis (at least five days a week) (50).
- Smoking status: current smoker – light (<10 cigarettes per day); current smoker – moderate (10-20 per day); current smoker – heavy (20+ per day); ex-regular smoker; non-smoker (never regularly smoked).
- Alcohol consumption frequency: almost every day; once or twice a week; once or twice a month/once every couple months; once or twice year; not at all/non-drinker.
- Health Status is measured by three variables
  ° (1) self-reported general health: (good; fair; poor)
  ° (2) limiting long standing illness (whether has an illness, disability or infirmity that has affected them over a period of time and limits their daily activities): yes; no
  ° (3) psychological health measured using General Health Questionnaire 12 (GHQ12) (51): score 0; score 1–3; score 4+.
- Marital status and presence of children in household: married/cohabiting, no children; married/cohabiting, with children; single/separated/divorced/widowed, no children; single/separated/divorced/widowed with children.

### Data analyses

The combined Health Survey for England and Census data have a hierarchical structure, i.e., individuals (level 1) live within areas (level 2). To deal with the hierarchical nature of the data, gender-specific random intercept multilevel modeling techniques were used to model the data using the Runmlwin command in Stata MP. Interaction models were run to formally test the effect of area deprivation and co-ethnic density respectively for each ethnic group compared with the White group. The models were run as follows:

Model 0: baseline variance components model, adjusted for hip circumference.
Model 1: age, ethnicity, and hip circumference.
Model 2: Model 1, plus all individual-level control variables.
Model 3: Model 2, plus area-deprivation.
Model 4: Model 2, plus area-deprivation and co-ethnic density.
Model 5: Model 3 with an area-deprivation and ethnicity interaction.
Model 6: Model 4 with a co-ethnic density and ethnicity interaction.

Predicted values for each ethnic group were then calculated.

Survey weights were used to account for the sampling design of the survey and for non-response. The Akaike Information Criterion (AIC) was calculated to explore the goodness-of-fit of the multi-level models (52). The White ethnic group was used as the reference category in the models because it has the largest sample and because this approach enables an exploration of the causal pathways that are relevant to the ethnic minority groups.

## Results

Table 1 shows the overall sample size for men and women within each ethnic group. The total sample size was 27,892, including 12,826 men and 15,066 women.

**Table 1:** Ethnic group sample sizes by gender^*^.

**Ethnic group**	**Men**	**Women**	**Total**
Black Caribbean	531	798	1,329
Black African	163	212	375
Indian	754	786	1,540
Pakistani	574	625	1,199
Bangladeshi	408	461	869
Chinese	373	431	804
Irish	670	876	1,546
White	9,353	10,877	20,230
Total	12,826	15,066	27,892

* Aged 16–74 with valid waist and hip measurement.

Table 2 shows the distribution of all control variables, and the IMD variable, by ethnic group. The Bangladeshi group is far more likely to live in the most deprived areas of the country than other ethnic groups (83%) followed by the Pakistani group (54%), Black Caribbean (48%) and Black African groups (44%). The White group is the least likely to live in the most deprived areas (15%), followed by Irish (19%), Indian (24%) and Chinese (24%).

**Table 2:** Distribution of control variables and IMD by ethnic group (weighted column proportions, unweighted N).

**Ethnic group**	**Black Caribbean**	**Black African**	**Indian**	**Pakistani**	**Bangladeshi**	**Chinese**	**Irish**	**White**	**Total**
**Mean age (years)**	41.7	35.6	40.7	35.5	35.7	39.9	45.1	45.0	43.6
N (unweighted)	1,329	375	1,540	1,199	869	804	1,546	20,230	27,892
**Social Class**									
Professional	2.3	3.7	6.1	4.6	1.4	7.8	6.2	4.6	4.5
Managerial/ technical/skilled non-manual	46.3	41.4	45.1	26.3	17.2	50.7	52.3	50.6	50.0
Manual	45.0	33.2	36.2	33.2	40.3	28.8	39.4	41.5	41.3
Never worked	2.2	12.1	6.8	27.6	30.7	6.5	1.4	1.6	2.2
Other	4.2	9.6	5.7	8.3	10.4	6.3	0.7	1.7	1.9
N (unweighted)	1,328	375	1,538	1,197	869	804	1,545	20,207	27863
**Qualifications**									
Degree level or above	10.3	27.6	27.4	14.2	7.8	21.3	19.2	14.6	14.9
Higher education below degree	15.6	10.7	6.6	4.1	1.6	13.0	11.1	11.0	10.9
A-level or equivalent	9.4	11.0	9.0	6.0	5.7	8.8	11.6	10.2	10.1
GCSE or equivalent	27.2	15.3	20.1	20.7	15.4	14.2	27.9	33.2	32.4
No qualifications	24.6	14.6	23.4	39.2	53.6	24.2	24.8	23.7	24.0
Full-time student	12.8	20.8	13.5	15.8	15.9	18.5	5.4	7.3	7.6
N (unweighted)	1,327	373	1,538	1,197	869	804	1,546	20,215	27869
**Equivalised household income**									
Quintile 1—lowest quintile	25.5	22.1	19.2	34.5	57.9	20.9	10.6	12.1	13.0
Quintile 2	17.1	15.4	18.3	21.6	11.3	15.7	13.5	16.0	16.0
Quintile 3	16.4	13.4	16.2	10.6	3.2	9.5	17.4	19.2	18.8
Quintile 4	14.8	20.1	11.5	5.0	1.9	13.4	20.5	21.9	21.1
Quintile 5—highest quintile	11.6	15.2	12.2	5.0	1.2	17.2	26.9	19.1	18.8
Missing income data	14.6	13.8	22.5	23.31	23.3	23.3	11.1	11.7	12.1
N (unweighted)	1,329	375	1,540	1,199	869	804	1,546	20,230	27892
**Migration status**									
UK born	51.0	23.4	24.7	30.9	13.3	20.0	76.6	100.00	95.7
Child migrant	15.7	14.4	20.6	21.8	30.2	16.9	8.0	0.0	1.3
Adult migrant (< 5 years)	2.6	18.9	6.9	6.8	7.0	5.0	0.5	0.0	0.4
Adult migrant (5–9 years)	1.9	11.9	5.8	9.2	11.8	7.4	0.4	0.0	0.4
Adult migrant (10–19 years)	1.8	20.8	10.0	10.8	17.8	14.6	3.1	0.0	0.6
Adult migrant (20+ years)	27.0	10.5	31.9	20.5	19.8	36.2	11.3	0.0	1.7
N (unweighted)	1,324	369	1,536	1,193	862	803	1,543	19,847	27477
**English language proficiency**									
None (does not read/speak)	0.0	1.3	4.0	8.2	23.2	12.4	0.0	0.0	0.4
Partial (reads or speaks)	2.5	8.8	2.9	8.1	12.3	6.8	0.0	0.0	0.3
Both (reads & speaks)	97.5	89.9	93.1	83.8	64.5	80.8	100.00	100.0	99.4
N (unweighted)	888	369	1,539	1,195	869	804	1,546	20,230	27440
**Dietary fat intake**									
Low fat	55.0	71.6	66.0	50.5	35.4	47.2	62.6	56.6	56.7
Medium fat	13.5	10.9	9.6	13.2	14.1	13.8	19.1	22.6	21.8
High fat	5.6	4.0	3.6	7.0	7.6	3.7	5.5	10.8	10.2
Missing fat data	25.9	13.4	20.8	29.4	42.9	35.2	12.7	10.1	11.3
N (unweighted)	1,329	375	1,540	1,199	869	804	1,543	20,230	27892
**Physical activity**									
High level	32.9	35.9	43.6	46.6	60.4	44.7	30.6	31.8	32.4
Low level	30.9	30.3	30.0	31.9	21.7	33.3	35.6	35.2	34.9
Medium level	36.2	33.8	26.4	21.5	17.9	22.0	33.8	33.0	32.7
N (unweighted)	1,325	374	1,538	1,196	866	804	1,546	20,210	27859
**Smoking status**									
Heavy smoker	3.1	0.5	2.2	3.1	3.4	1.8	9.0	9.3	9.0
Moderate smoker	10.2	6.6	4.3	6.8	8.8	4.7	11.8	11.5	11.3
Light smoker	15.0	10.0	6.8	6.5	12.5	6.0	8.8	7.2	7.4
Ex-smoker	14.8	8.3	7.3	4.8	5.5	9.2	27.5	25.0	24.2
Never smoked	56.9	74.6	79.4	78.8	69.8	78.2	42.8	47.0	48.2
N (unweighted)	1,317	369	1,534	1,180	849	800	1,544	20,173	27766
**Alcohol consumption**									
Almost every day	29.8	18.6	16.0	2.2	1.5	16.3	31.6	31.6	30.7
Once or twice a week	28.2	25.1	17.5	2.1	0.3	24.8	17.5	18.1	18.0
Once or twice month/once every couple months	8.2	10.6	7.7	1.2	0.1	13.3	4.6	6.0	6.0
Once or twice a year	14.6	35.4	45.4	92.9	97.4	32.5	8.2	6.2	8.7
None in last 12 months/non-drinker	19.2	10.3	13.5	1.6	0.8	13.1	38.1	38.0	36.6
N (unweighted)	1,319	369	1,531	1,176	839	802	1,543	20,183	27762
**General health status**									
Good	67.4	80.5	68.6	66.3	56.8	76.2	77.5	77.8	77.2
Fair	24.1	14.0	23.2	20.5	26.2	19.4	16.8	17.0	17.2
Poor	8.5	5.5	8.2	13.2	17.0	4.4	5.8	5.3	5.5
N (unweighted)	1,326	375	1,539	1,198	869	804	1,546	20,228	27885
**Limiting longstanding illness**									
Yes	26.1	14.3	21.2	24.8	28.2	11.7	24.8	23.3	23.3
No	73.9	85.7	78.8	75.2	71.8	88.3	75.2	76.7	76.7
N (unweighted)	1,328	374	1,540	1,199	869	804	1,546	20,277	27887
**GHQ12**									
Score 0	50.3	55.7	54.9	52.7	45.4	62.7	60.3	61.6	61.2
Score 1-3	31.8	28.0	28.4	27.4	29.3	29.7	23.5	24.4	24.6
Score 4+	17.9	16.4	16.6	19.9	25.4	7.6	16.2	14.0	14.2
N (unweighted)	1,231	325	1,401	943	640	740	1,488	19,610	26378
**Marital status/children in household**									
Married/cohabiting, no children	31.0	18.0	36.8	15.4	9.8	33.1	49.5	50.0	48.8
Married/cohabiting, children	28.5	38.4	43.1	63.4	69.8	42.0	31.8	26.9	28.0
Single/separated/divorced/widowed, no children	21.5	22.03	14.7	7.5	4.9	17.7	14.8	16.9	16.7
Single/separated/divorced/widowed, with children	19.1	21.5	5.4	13.7	15.5	7.2	3.9	6.2	6.5
N (unweighted)	1,328	375	1,540	1,198	869	804	1,545	20,228	27887
**Area-deprivation**									
IMD 1 – least deprived	3.2	3.7	13.0	2.7	0.4	14.8	20.8	22.9	22.0
IMD 2	5.8	9.2	17.2	5.3	1.2	15.8	21.5	21.1	20.5
IMD 3	15.1	13.3	19.6	12.0	2.0	18.9	18.4	20.4	20.0
IMD 4	27.3	29.6	26.8	26.0	13.8	26.2	19.9	20.2	20.5
IMD 5 – most deprived	48.5	44.3	23.6	54.0	82.7	24.3	19.4	15.3	16.9
N (unweighted)	1,329	375	1,540	1,199	869	804	1,546	20,230	27892
**Mean hip (centimeters)**	104.9	107.4	101.2	102.5	96.8	96.8	104.7	104.6	104.4
N (unweighted)	1,329	375	1,540	1,199	869	804	1,546	20,230	27892

Table 3 shows summary statistics for co-ethnic density by ethnic group and by gender. The White group has, by far, the largest level of co-ethnic density, ranging from 6.3 to 100% for men and 7.7 to 100% for women (and with a mean of 91% co-ethnic density for both men and women). The Pakistani and Bangladeshi groups also have large levels of co-ethnic density. For example, Pakistani women's co-ethnic density ranges from 0 to 73.7%, with a mean of 19.8%. The Chinese and the Irish groups have, by far, the lowest levels of co-ethnic density. Chinese co-ethnic density ranges from 0 to 9.2%, with a mean of 1.3%, while, for example, Irish men's co-ethnic density ranges from 0 to 10.8% with a mean of 2.0%. The Black Caribbean, Black African and Indian groups also have fairly low levels of co-ethnic density. For example, for Black Caribbean men and women co-ethnic density ranges from 0 to 29.1% with a mean of 8.9% for men and 9.4% for women. It is possible that the low-range for some ethnic groups, particularly the Irish and Chinese, may affect the results.

**Table 3:** Co-ethnic density (continuous variable) by ethnic group by gender: summary statistics^*^.

	**Mean**	**Range**		**Percentiles**			**N (unweighted)**
		**Min**	**Max**	**25%**	**50%**	**75%**	
**Men**							
Black Caribbean	8.9	0.0	29.1	3.3	7.4	13.2	531
Black African	6.8	0.0	33.6	1.44	4.0	9.1	163
Indian	16.9	0.0	69.3	3.8	9.6	24.3	754
Pakistani	18.0	0.0	73.7	3.5	10.1	29.8	574
Bangladeshi	21.1	0.0	61.3	3.2	12.2	34.9	408
Chinese	1.3	0.0	9.2	0.6	1.1	1.7	373
Irish	2.0	0.0	10.8	0.9	1.7	2.6	670
White	90.8	6.3	100.00	89.0	94.3	97.9	9,353
**Women**							
Black Caribbean	9.4	0.0	29.1	3.9	8.9	13.7	798
Black African	6.3	0.0	26.5	1.4	4.0	8.9	212
Indian	16.5	0.0	69.3	3.9	9.7	22.3	786
Pakistani	19.8	0.0	73.7	4.4	11.2	33.3	625
Bangladeshi	22.2	0.0	61.3	4.4	14.5	35.6	461
Chinese	1.3	0.0	9.2	0.6	1.1	1.8	431
Irish	1.9	0.0	11.7	0.8	1.5	2.5	876
White	90.9	7.7	100.0	89.0	94.3	98.0	10,877

* Aged 16–74 with valid waist and hip measurement.

Table 4 shows the results of the baseline variance components model, for all cases. The intra-class correlation (ICC) (the proportion of the variance that is attributed to the area-level) shows that the values of waist circumference are correlated within areas by 0.11 (women) and 0.06 (men).

**Table 4:** Baseline Variance Components model—waist circumference (adjusted for hip circumference), all cases.

	**Area variance**	**Individual variance**	**ICC**
	**(95% CI)**	**(95% CI)**	
**Women**	5.60 (4.74, 6.45)	45.41 (43.83, 46.99)	0.11
**Men**	3.07 (2.26, 3.87)	45.62 (44.13, 47.10)	0.06

Tables 5, 6 show the results for Models 1–4. The tables show the change to the waist circumference of each ethnic group, when the extra variables are entered into the models (Table 5 for women and Table 6 for men). The largest change to the coefficients occurs between Models 1 and 2 i.e., when the individual level variables are entered into the model. Tables 5, 6 show that when area deprivation is entered into Model 3, waist circumference decreases, relative to the White group, for men and women in all ethnic groups when compared with Model 2 [though not all results are statistically significant (*p* < 0.05)]. In model 4, when co-ethnic density is entered into the model, the changes to the waist circumference coefficients reflect an increase in men and women's waist, on average, for all ethnic minority groups relative to White men and women [though not all results are statistically significant (*p* < 0.05)]. The changes to the waist circumference coefficients between Models 3 and 4 are very small for women's waist but the changes for men's waist are larger.

**Table 5:** Ethnic differences in waist circumference (cm) compared with White British (women).

**Women**	**Model 1 (adjusted for age, hip, ethnicity)**		**Model 2 (Model 1** + **all individual level variables)**		**Model 3 (Model 2** + **IMD)**		**Model 4 (Model 2** + **IMD** + **co-ethnic density)**	
**Ethnic group**	**B**	**95% CI**	**B**	**95% CI**	**B**	**95% CI**	**B**	**95% CI**
Black Caribbean	2.56**	(2.03, 3.09)	1.64**	(0.96, 2.31)	1.39**	(0.71, 2.07)	1.52**	(0.46, 2.57)
Black African	1.63**	(0.50, 2.77)	0.76	(−0.59, 2.11)	0.50	(−0.85, 1.86)	0.63	(−0.95, 2.21)
Indian	2.09**	(1.54, 2.64)	0.64	(−0.04, 1.32)	0.56	(−0.12, 1.24)	0.68	(−0.38, 1.73)
Pakistani	4.54**	(3.88, 5.19)	2.18**	(1.34, 3.03)	1.99**	(1.14, 2.84)	2.11**	(0.98, 3.23)
Bangladeshi	6.99**	(6.34, 7.65)	4.02**	(2.99, 5.04)	3.73**	(2.68, 4.77)	3.83**	(2.62, 5.04)
Chinese	2.64**	(2.02, 3.26)	1.59**	(0.79, 2.40)	1.53**	(0.73, 2.34)	1.67**	(0.46, 2.88)
Irish	1.40**	(0.85, 1.95)	0.84**	(0.27, 1.40)	0.75*	(0.18, 1.31)	0.88	(−0.17, 1.94)

* P < 0.05;

** P < 0.01; N = 15,066 (Model 1); 13,645 (Models 2–4).

AIC: model 1 99,746; model 2 89,437; model 3 89,423; model 4 89,425.

**Table 6:** Ethnic differences in waist circumference (cm) compared with White British (men).

**Men**	**Model 1 (adjusted for age, hip, ethnicity)**		**Model 2 (Model 1** + **all individual level variables)**		**Model 3 (Model 2** + **IMD)**		**Model 4 (Model 2** + **IMD** + **co-ethnic density)**	
**Ethnic group**	**B**	**95% CI**	**B**	**95% CI**	**B**	**95% CI**	**B**	**95% CI**
Black Caribbean	−2.36**	(−2.86, −1.86)	−3.07**	(−3.73, −2.42)	−3.27**	(−3.93, −2.61)	−2.90**	(−3.89, −1.92)
Black African	−2.19**	(−3.14, −1.25)	−2.79**	(−3.89, −1.70)	−3.01**	(−4.12, −1.90)	−2.64**	(−3.96, −1.31)
Indian	2.42**	(1.95, 2.90)	1.47**	(0.88, 2.07)	1.40**	(0.80, 2.01)	1.75**	(0.85, 2.65)
Pakistani	2.28**	(1.76, 2.80)	0.77*	(0.01, 1.54)	0.59	(−0.19, 1.37)	0.93	(−0.08, 1.94)
Bangladeshi	3.33**	(2.65, 4.02)	0.57	(−0.32, 1.46)	0.35	(−0.56, 1.25)	0.66	(−0.41, 1.72)
Chinese	−0.33	(−0.88, 0.23)	−0.77*	(−1.47, −0.07)	−0.84*	(−1.54, −0.15)	−0.44	(−1.49, 0.62)
Irish	0.85**	(0.36, 1.33)	0.45	(−0.04, 0.94)	0.38	(−0.12, 0.87)	0.77	(−0.15, 1.70)

* P < 0.05;

** P < 0.01; N = 12,826 (Model 1); 11,761 (Models 2–4).

AIC: model 1 81,806; model 2 74,308; model 3 74,291; model 4 74,293.

**Figure 1:**
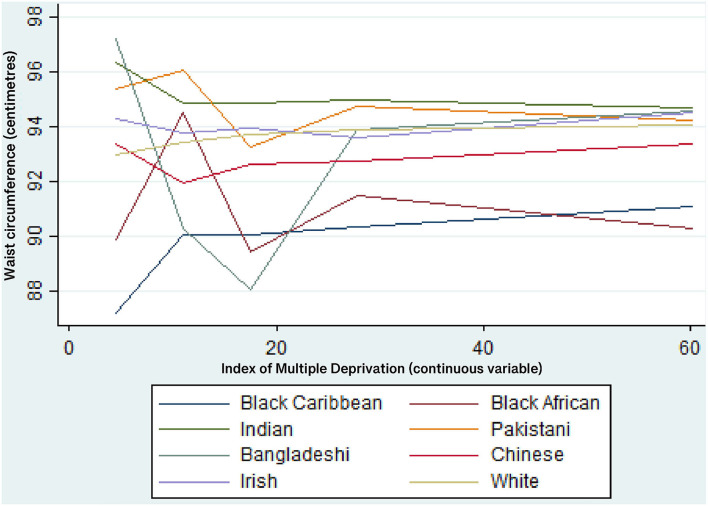
Predicted values of waist circumference (cm) for each ethnic group—men. Multi-level linear regression models adjusted for all individual-level variables, IMD, co-ethnic density and an ethnic group*IMD interaction.

Table 7 shows the results from Model 5, where ethnic group and IMD interactions are entered into the models. The results show that only 12 of the 56 ethnicity-IMD interactions are statistically significant (*p* < 0.05). This suggests that the impact of area deprivation is mostly equivalent across each ethnic group. Also, the inclusion of the interaction terms does not improve the goodness-of-fit (AIC) of the models when compared Models 1-4. There are only two statistically significant interactions for women, for Indian in IMD3 and Pakistani women in IMD2. This suggests that the relationship between area deprivation and waist circumference does not vary by ethnicity, on average, for women. For men, there are ten statistically significant interactions in total, for Black Caribbean men in IMD5, Indian men in IMD2-5, Bangladeshi men in IMD2-5 and Irish men in IMD4. The results for Indian and Bangladeshi men are consistent across 4 out of the 5 IMD groups. Figure 1 illustrates the predicted values of men's waist for each ethnic group as area deprivation increases (using the continuous IMD variable). It shows that waist circumference (cm) for Indian and Bangladeshi men decreases as area deprivation increases, on average, compared with an increase for White men's waist circumference. The equivalent chart for women is not shown as the results from Table 7 show that, for women, the impact of area deprivation is mostly equivalent across each ethnic group.

**Table 7:** Multi-level linear regression: ethnic group and IMD interactions.

**Waist (cm)**	**Women**		**Men**	
**Interactions**	**B**	**95% CI**	**B**	**95% CI**
Black Caribbean * IMD 2	0.24	(−3.85, 4.34)	2.38	(−2.12, 6.88)
Black Caribbean * IMD 3	0.50	(−2.79, 3.81)	2.11	(−0.92, 5.13)
Black Caribbean * IMD 4	2.01	(−1.33, 5.35)	2.16	(−0.64, 4.96)
Black Caribbean * IMD 5	1.38	(−1.86, 4.61)	2.73*	(0.06, 5.41)
Black African * IMD 2	2.22	(−3.20, 7.63)	4.17	(−1.36, 9.69)
Black African * IMD 3	1.46	(−2.74, 5.66)	−0.97	(−5.81, 3.87)
Black African * IMD 4	−0.12	(−4.66, 4.43)	0.55	(−3.79, 4.90)
Black African * IMD 5	1.80	(−1.94, 5.53)	−0.80	(−4.97, 3.36)
Indian * IMD 2	−1.66	(−3.62, 0.30)	−1.97**	(−3.47, −0.48)
Indian * IMD 3	−2.49**	(−4.32, −0.66)	−2.23**	(−3.52, −0.94)
Indian * IMD 4	−0.97	(−2.59, 0.65)	−2.42**	(−3.80, −1.03)
Indian * IMD 5	−1.38	(−3.23, 0.47)	−2.82**	(−4.43, −1.20)
Pakistani * IMD 2	3.76*	(0.28, 7.23)	0.16	(−3.00, 3.32)
Pakistani * IMD 3	1.44	(−2.01, 4.89)	−2.82	(−6.07, 0.42)
Pakistani * IMD 4	1.62	(−1.62, 4.86)	−1.67	(−4.67, 1.32)
Pakistani * IMD 5	1.99	(−1.19, 5.17)	−2.49	(−5.36, 0.37)
Bangladeshi * IMD2	−0.66	(−8.71, 7.40)	−7.00**	(−10.23, −3.77)
Bangladeshi * IMD 3	−1.73	(7.80, 4.34)	−9.74**	(−14.85, −4.63)
Bangladeshi * IMD 4	1.26	(−0.84, 3.36)	−3.95**	(−6.98, −1.70)
Bangladeshi * IMD 5	0.38	(−0.92, 1.69)	−3.95**	(−5.69, −2.20)
Chinese * IMD 2	−0.49	(2.26, 1.29)	−1.82	(−3.73, 0.09)
Chinese * IMD 3	−1.43	(−3.22, 0.36)	−1.41	(−3.44, 0.61)
Chinese * IMD 4	−0.75	(−2.63, 1.13)	−1.50	(−3.36, 0.36)
Chinese * IMD 5	−1.13	(−3.22, 0.96)	−0.98	(−3.01, 1.05)
Irish * IMD 2	−1.04	(−2.71, 0.64)	−0.95	(−2.52, 0.61)
Irish * IMD 3	0.49	(−1.15, 2.13)	−1.19	(−2.68, 0.31)
Irish * IMD 4	−0.40	(−2.03, 1.23)	−1.63*	(−3.04, −0.21)
Irish * IMD 5	0.18	(1.36, 1.73)	−0.93	(−2.32, 0.45)
N	13645		11761	

* P < 0.05;

** P < 0.01 AIC: women 89,408; men 74,295.

Adjusted for all individual-level variables, IMD and ethnic group*IMD interaction.

Table 8 shows that the results from Model 6. where ethnic group and co-ethnic density interactions are entered into the models. The results show that only 2 of the 14 ethnicity-co-ethnic density interactions are statistically significant at *p* < 0.05 (for Black Caribbean women and Indian men). The inclusion of the interaction terms does not improve the AIC for the models for men or women. Given that only one of the seven interaction terms are significant for each gender, this suggests that the relationship between co-ethnic density and waist circumference is mostly equivalent across each ethnic group.

**Table 8:** Multi-level linear regression: ethnic group and co-ethnic density interactions (waist).

**Waist (cm)**	**Women**		**Men**	
**Interactions**	**B**	**95% CI**	**B**	**95% CI**
Black Caribbean*co-ethnic density	0.11*	(0.00, 0.21)	−0.01	(−0.12, 0.09)
Black African*co-ethnic density	0.05	(−0.11, 0.20)	−0.07	(−0.19, 0.05)
Indian*co-ethnic density	−0.02	(−0.06, 0.02)	−0.04**	(−0.07, −0.02)
Pakistani*co-ethnic density	0.02	(−0.02, 0.06)	−0.02	(−0.06, 0.01)
Bangladeshi*co-ethnic density	0.00	(−0.04, 0.04)	0.00	(−0.04, 0.03)
Chinese*co-ethnic density	−0.44	(−0.89, 0.01)	−0.03	(−0.59, 0.53)
Irish*co-ethnic density	0.00	(−0.24,0.25)	−0.06	(−0.32, 0.19)
N	13,645		11,761	

* P < 0.05;

** P < 0.01; AIC: women 89,427; men 74,288.

Adjusted for all individual-level variables, IMD, co-ethnic density and an ethnic group*co-ethnic density interaction.

**Figure 2:**
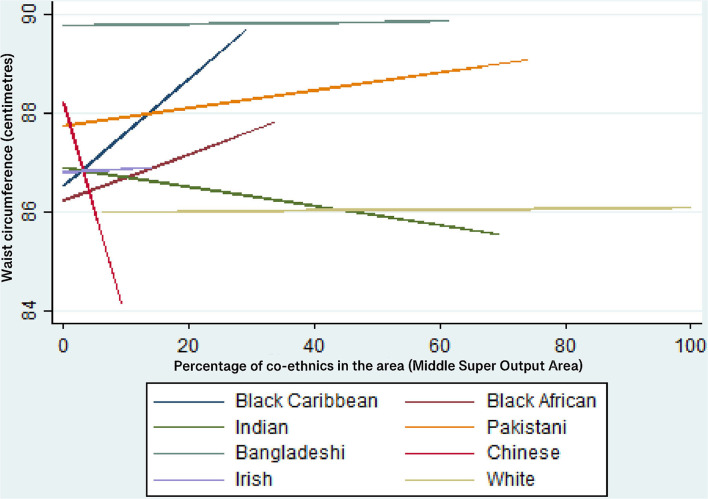
Predicted values of waist circumference (cm) for each ethnic group—women. Multi-level linear regression models adjusted for all individual-level variables, IMD, co-ethnic density and an ethnic group*co-ethnic density interaction.

**Figure 3:**
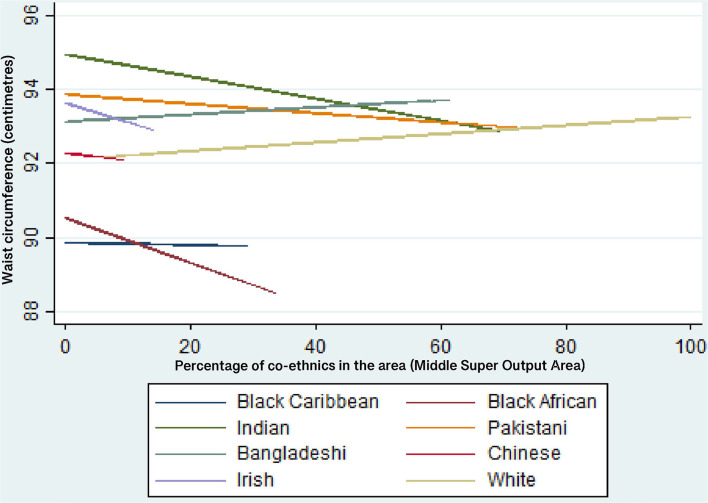
Predicted values of waist circumference (cm) for each ethnic group—men. Multi-level linear regression models adjusted for all individual-level variables, IMD and an ethnic group*IMD interaction.

Figure 3 illustrate the predicted values of waist for each ethnic group as co-ethnic density increases, for women and men respectively. As noted in Table 8, only the results for Black Caribbean women and Indian men are statistically significant. Figure 2 shows that Black Caribbean women's waist circumference increases as co-ethnic density increases, on average. Figure 3 shows that Indian men's waist circumference decreases as co-ethnic density increases, on average.

## Discussion

This research adds new results to the study of ethnic differences in obesity by addressing the following research question: Does the relationship between co-ethnic density/area deprivation and waist circumference differ by ethnic group in England?

The research is novel because there have been no studies in the UK that have explored the relationship between *central obesity* and co-ethnic density/area-level deprivation for different ethnic groups. This is an important addition to the current literature on ethnic differences in obesity because central obesity is important for South Asian ethnic groups who have an increased risk of developing the metabolic syndrome (a group of risk factors for heart disease and other health problems such as diabetes) (2, 3). The research is also novel because it presents the results at a national level for England, (using HSE and Census data) and because it includes a larger number of ethnic minority groups than other UK studies [e.g., (29)]. These are the most up to date nationally representative data available; there have been no UK studies since that study *central obesity* (rather than BMI) and have a nationally representative sample of ethnic minorities.

Regarding research question 1, the results suggest that the relationship between co-ethnic density and waist circumference is *mostly* equivalent across ethnic groups. However, the statistically significant result that Black Caribbean women's waist circumference increases as co-ethnic density increases is important because Black Caribbean women have a higher risk of central obesity than the general population in England. This result also supports the results of authors such as (53, 54) which show that increased co-ethnic density may have a detrimental effect on Black Caribbean health. Previous research suggests that this is related to lower levels of social cohesion in areas of higher Black Caribbean ethnic density; it has been hypothesized that the weaker social cohesion of the Black Caribbean group is related to the internalizing of negative racialized stereotypes portrayed in the media (23, 53, 55). Another potential mechanism for the co-ethnic density effect is the social norms theory i.e., that behavioral norms within ethnic groups may be stronger/more prevalent within areas of high co-ethnic density (53). Previous research and theory suggest that larger body sizes are viewed more positively among Black Caribbean women (56) and such social norms may be amplified in areas of higher co-ethnic density. However, this theory is heavily contested because there is evidence to suggest that Black African, Black Caribbean and South Asian ethnic minority women living in the UK have similar body size ideals to those of the White majority (slimness) rather than ideals of larger body sizes (57–60). Attitudes to body size are not tested in this research so it is important that conclusions are not drawn from this research in relation to body size ideals when there is no evidence to support such claims. It is also important to note that the same effect of co-ethnic density is not observed for Black Caribbean men in this study. This suggests that the association between social cohesion and ethnic density works differently for Black Caribbean men than for Black Caribbean women and this is an area of research that would benefit from further exploration.

Also, the statistically significant result for Indian men (a decrease in waist circumference as co-ethnic density increases) is important because the Indian ethnic group have an increased risk of developing the metabolic syndrome. This result could be due to higher levels of co-ethnic density having a buffering effort on exposure to racial discrimination and interpersonal racism and by providing access to larger social networks and increase increased social support/cohesion, as suggested by other research (23). With regard to the results for other ethnic groups, for both men and women, it has been noted that null associations between ethnic density and health in the UK are likely to be the result of a lack of statistical power limited by narrow ranges of ethnic density and by small samples of each ethnic subgroup (61).

With relation to research question 2, the results suggest that, for women, the relationship between area deprivation and waist circumference does not vary by ethnicity. However, for men, the results for Indian and Bangladeshi men are statistically significant and the predicted values suggest that waist circumference (cm) decreases as area deprivation increases, on average, compared with an increase for White men's waist circumference. This is an important result because Indian and Bangladeshi men are identified as being at increased risk of developing the metabolic syndrome. Therefore, this needs further exploration as to why Indian and Bangladeshi men may be protected from the detrimental effects of area-level deprivation upon central obesity. There are many potential hypotheses for these results such as theories of protective behavioral norms and increased social support, but more direct research is needed to explore such hypotheses.

The results have also highlighted gender differences in waist circumference within ethnic groups, even after controlling for many other variables (Table 5). For example, Black Caribbean women, on average, have a higher waist circumference than White women but Black Caribbean men, on average, have a lower waist circumference than white men, even after the full controls. This is an interesting result and needs further exploration into the mechanisms behind these gender differences. There are also gender differences in terms of the association between waist circumference and co-ethnic density that require further exploration. The same effect of co-ethnic density on Black Caribbean women is not observed for Black Caribbean men and the causal pathways behind the different results for Black Caribbean men and women is an area for further research.

Within the UK, there is only one study (29) that has explored the relationship between co-ethnic density and obesity; the study explored the relationship between BMI (not central obesity) and co-ethnic density for five ethnic groups in East London (not on a national level). The study reported that an increase in co-ethnic density was associated with a decrease in BMI for Indian women but with an increase in BMI for Black African men and women. These results were not replicated in our study but this is likely to be due to the different outcome measure and the different geographical coverage.

## Limitations

The cross-sectional design of the HSE limits the degree to which causal pathways can be determined. However, a longitudinal dataset with a large enough sample of adult ethnic minorities and an adequate array of variables to track the pathways to obesity, over time, does not exist in the UK.

The data used in this study are old which means that the levels of obesity within ethnic groups may have changed due to, for example, changes in the age and generation profiles of ethnic groups and increased social mobility among some ethnic groups. However, the theorized area-level pathways to obesity are considered unlikely to have changed substantially and this research is an exploration of the area-level pathways to ethnic differences in obesity.

There are a number of unmeasured elements within this research. For instance, local access to food outlets or green space, experiences of racism or discrimination, stress, allostatic load, the influence of early life exposures and perceptions of body image and stigma (61).

There is a low range of co-ethnic density for some ethnic groups, particularly the Chinese and Irish groups. This lack of variation may affect the results.

The use of MSOA (or any other artificial geographical boundary) limits the exploration of area effects because it does not take into consideration exposure to neighborhoods outside of the residential MSOA (*via* activities such as employment, social activities or shopping). However, (59) note that the optimum geographical level for measuring the effects of ethnic density is undecided. MSOA was considered an adequate geographical level for this study because MSOAs are large enough to include nearby shopping facilities, leisure facilities and greenspace. The boundaries used in geographical administrative data may not adequately capture the lived experience of people within an area (61). Perceived ethnic density may, therefore, be a more accurate measure of the strength of co-ethnic contact and identification within an area (62).

There were no data available on how long the participants had been living at their current address. It is, also, not possible to determine whether the results are partially due to selection effects. There may be area-level selection effects—an individual may select to live in (or stay in) an area rather than being randomly distributed into an area (63). Additionally, the HSE sample design involves clustering at the household level so it is possible that some of the area-level variance reported in the results are attributable to the household level or individual level. To test this hypothesis an exploratory three-level variance component model, including household-level, was run for the waist circumference outcome for men and women separately (results not shown). The results suggested that household-level variance has very little effect on the area or individual variance for men. For women, there was a small amount of the area-level variance attributed to the household level but there was a larger effect on the individual-level variance. However, it is possible that the effect on individual-level variance may largely be a consequence of selection into the household level.

It was not possible to explore the ethnic variance in waist circumference at the area level because the small sample sizes for some ethnic groups in this study did not support these analyses. If a dataset with a larger ethnic minority group sample were to become available, this could be explored in future research.

## Conclusions

Central obesity is a risk factor in many health-related outcomes, and is particularly important for the Indian, Pakistani and Bangladeshi groups who have an increased risk of developing the metabolic syndrome. The results of this study suggest that co-ethnic density and area-deprivation are both protective against increased waist circumference for Indian men and that area deprivation is protective for increased waist circumference for Bangladeshi men. Further research is needed to understand the mechanisms through which these results might arise.

Black Caribbean women have a higher risk of central obesity than the general population in England. The result that an increase in co-ethnic density is associated with an increase in waist circumference for Black Caribbean women adds knowledge to the field of potential pathways to the higher risk of central obesity for Black Caribbean women. It also supports the results of other research suggesting that co-ethnic density is detrimental to Black Caribbean health in the UK (54, 55). Further research is needed to understand the mechanism through which increasing co-ethnic density is associated with an increase in waist circumference for Black Caribbean women.

## Data availability statement

Publicly available datasets were analyzed in this study. The Health Survey for England Data (1998, 1999, 2003, and 2004) are available from the UK Data Service under End User Licence conditions: National Centre for Social Research, University College London. Department of Epidemiology and Public Health, 2010, Health Survey for England, 1998, 1999, 2003, and 2004 [data collection], UK Data Service. Retrieved from https://discover.ukdataservice.ac.uk/series/?sn=2000021. The small-area Census data that are linked to the Health Survey for England data were obtained from the National Centre for Social Research with permission from NHS-Digital. Permission has to be sought from NHS-Digital to use these data.

## Ethics statement

The studies involving human participants were reviewed and approved by an Independent Research Ethics Committee. Ethical approval was obtained as follows: 1998 HSE survey: North Thames Multi-Centre Research Ethics Committee (MREC) and from all Local Research Ethics Committees (LRECs) in England. Approval for the revised blood protocol for minors was obtained from LRECs covering the Primary Sampling Units in which the protocol was adopted. 1999 HSE survey: North Thames Multi-Centre Research Ethics Committee and from all Local Research Ethics Committees (LRECs) in England. 2003 and 2004HSE surveys: LondonMulti-Centre Research Ethics Committee (MREC). All relevant Local Research Ethics Committees were informed about the survey. Verbal consent was obtained from adult participants. Verbal consent was documented by the survey field interviewers. The data do not include minors. All data are fully anonymized before they were accessed from the UK Data Service. I do not have information on who witnessed the verbal consent as this is secondary data. It was approved by the Ethics Board. The patients/participants provided their written informed consent to participate in this study.

## Author contributions

VH is the sole author, devised the study, conducted the analyses, and wrote the paper.

## Funding

The Economic and Social Research Council funded the initial research that led to the development of this paper. The open access fees are paid by the University of Manchester (Grant No. ES/F003285/1).

## Conflict of interest

The author declares that the research was conducted in the absence of any commercial or financial relationships that could be construed as a potential conflict of interest.

## Publisher's note

All claims expressed in this article are solely those of the authors and do not necessarily represent those of their affiliated organizations, or those of the publisher, the editors and the reviewers. Any product that may be evaluated in this article, or claim that may be made by its manufacturer, is not guaranteed or endorsed by the publisher.
